# Does the Anastomosis Recipient Vessel Have an Influence on Free Flap Perfusion in Microvascular Head and Neck Reconstruction—A Retrospective Analysis of 338 Cases with Comparison of Flap Perfusion between Different Arterial and Venous Recipient Vessels in Radial Free Forearm Flaps, Anterolateral Thigh Flaps, and Fibula Free Flaps

**DOI:** 10.3390/jcm13102763

**Published:** 2024-05-08

**Authors:** Mark Ooms, Philipp Winnand, Marius Heitzer, Marie Sophie Katz, Florian Peters, Johannes Bickenbach, Frank Hölzle, Ali Modabber

**Affiliations:** 1 Department of Oral and Maxillofacial Surgery, University Hospital RWTH Aachen, Pauwelsstraße 30, 52074 Aachen, Germany; 2 Department of Intensive Care Medicine, University Hospital RWTH Aachen, Pauwelsstraße 30, 52074 Aachen, Germany

**Keywords:** microvascular head and neck reconstruction, microsurgery, free flap, anastomosis recipient vessel, perfusion, blood flow, oxygen saturation, surgical outcomes

## Abstract

**Background:** Flap perfusion is a prerequisite for microvascular free flap survival and a parameter routinely used for flap monitoring. The aim of this study was to investigate the influence of the anastomosis recipient vessel on flap perfusion. **Methods:** Flap perfusion was retrospectively analyzed in 338 patients who underwent head and neck reconstruction with microvascular free flaps between 2011 and 2020. The Oxygen-to-see tissue oxygen analysis system measurements for intraoperative and postoperative flap blood flow, hemoglobin concentration, and hemoglobin oxygen saturation at 8 and 2 mm tissue depths were compared between arterial anastomosis recipient vessels (external carotid artery [ECA], facial artery [FAA], lingual artery [LIA], and superior thyroid artery [STA]) and venous anastomosis recipient vessels (internal jugular vein [IJV], combination of IJV and IJV branches, IJV branches, and external jugular vein). **Results:** The postoperative hemoglobin concentration at 2 mm tissue depth differed significantly between arterial anastomosis recipient vessels (ECA, 41.0 arbitrary units [AU]; FAA, 59.0 AU; LIA, 51.5 AU; STA, 59.0 AU; *p* = 0.029). This difference did not persist in the multivariable testing (*p* = 0.342). No other differences in flap blood flow, hemoglobin concentration, or hemoglobin oxygen saturation were observed between the arterial and venous anastomosis recipient vessels (*p* > 0.05 for all). **Conclusions:** The arterial and venous recipient vessels used for anastomosis did not influence microvascular free flap perfusion. This underlines the capability of the studied recipient vessels to adequately perfuse free flaps, may explain the observed indifferent flap survival rates between commonly used anastomosis recipient vessels, and implies that the recipient vessel is not a confounding variable for flap monitoring with the Oxygen-to-see tissue oxygen analysis system. Further prospective studies are needed to confirm the findings.

## 1. Introduction

Free tissue transfer with microvascular free flaps is currently the standard method for reconstruction of the head and neck region, with the selection of arterial and venous recipient vessels for performing anastomosis with the flap pedicle vessels as an essential step in restoring flap perfusion [1,2,3,4,5,6]. 

Free flap perfusion is essential for flap viability and thus for flap survival, as studies have already shown that decreased flap perfusion leads to flap failure [7,8,9,10,11,12]. Free flap perfusion is therefore frequently used as a parameter for postoperative flap monitoring [8,9,10]. Interestingly, commonly used arterial recipient vessels such as the external carotid artery (ECA), facial artery (FAA), lingual artery (LIA), and superior thyroid artery (STA), differ in their vessel diameters [13,14,15,16,17]. Furthermore, commonly used venous recipient vessels, such as the internal jugular vein (IJV), internal jugular vein branches (IJVB), and external jugular vein (EJV), differ in their vessel diameters and the extents of their respiratory venous pump effects [18,19]. With regard both to the relationship between vessel diameter, resistance, and blood flow, as well as to the support of venous flap drainage by the respiratory pump effect, it is conceivable that the anastomosis recipient vessel diameter and the extent of the respiratory pump effect may have an influence on flap perfusion in terms of blood flow, hemoglobin oxygen saturation, and hemoglobin concentration [20,21]. Theoretically, a larger vessel diameter of the anastomosis recipient vessel should support blood inflow and outflow in the free flap due to lower vascular resistance, resulting in higher flap blood flow and hemoglobin oxygen saturation [8,10,18,20]. In addition, a more pronounced respiratory venous pump effect in the anastomosis recipient vessel, related to pleural negative pressure-induced dilation of the thoracic vessels and a consequently reduced central venous pressure inducing venous blood from the extrathoracic vessels into the thoracic vessels, should support venous drainage of the free flap, resulting in a lower flap hemoglobin concentration [8,10,19,21]. 

However, no study has examined the influence of anastomosis recipient vessels on flap perfusion to gain insight into microvascular free flap perfusion, to evaluate whether differences in flap failure rates between anastomosis recipient vessels might be attributable to differences in flap perfusion, and to clarify whether the arterial and venous anastomosis recipient vessels should be considered as confounding variables in flap perfusion monitoring [3,8,9,10,18,22,23,24,25]. Although free flap surgery is routinely performed, little is known to date about the physiology of free flap perfusion and the pathophysiology of free flap failure, respectively [23,24]. In addition, many studies have addressed the identification of risk factors for flap failure, with partially divergent results regarding the choice of the recipient vessel, whereby the influence of the anastomosis recipient vessel on flap perfusion is a potential but unconsidered link between recipient vessel choice and flap failure [3,6,8,9,10,14,18,19,22,25]. Moreover, for flap monitoring with the Oxygen-to-see tissue oxygen analysis system, threshold values indicating vascular flap compromise have been established with absolute values for blood flow and hemoglobin oxygen saturation, with the influence of the anastomosis recipient vessel on flap perfusion being a potential but unconsidered factor affecting their validity [8,9,10].

Therefore, the aim of this study was to investigate the influence of the arterial and venous anastomosis recipient vessel on microvascular free flap perfusion in head and neck reconstruction by comparing flap perfusion between different arterial and venous anastomosis recipient vessels in terms of blood flow, hemoglobin concentration, and hemoglobin oxygen saturation. 

## 2. Materials and Methods

### 2.1. Study Population

This study was conducted in accordance with the Declaration of Helsinki, and the protocol was approved by the local ethics committee of the Medical Faculty RWTH Aachen University (EK 309-20).

The study population consisted of 338 patients who underwent microvascular head and neck reconstruction with a radial free forearm flap (RFFF), anterolateral thigh flap (ALTF), or fibula free flap (FFF) for malignant or nonmalignant diseases in our Department of Oral and Maxillofacial Surgery between 2011 and 2020. The inclusion criteria were complete data records; age greater than 18; arterial anastomosis with the ECA, FAA, LIA, or STA as the arterial anastomosis recipient vessel; and venous anastomosis with the IJV, IJV and IJVB in combination (IJV + IJVB [IJVB: facial vein, superior thyroid vein, lingual vein, hypoglossal vein or retromandibular vein]), IJVB (facial vein or superior thyroid vein), or EJV as the venous anastomosis recipient vessel (Table 1). 

The data were obtained from clinical records. Comorbidities were recorded according to the discipline-specific guidelines. Smoking status was defined in accordance with commonly used definitions as actual or past daily smoking for a period of at least six months at the time of the pre-anesthesia interview [26]. The status of prior neck dissection was defined as positive in patients who had undergone neck dissection, including an anatomic dissection of the recipient vessel prior to the actual surgery. The status of prior neck irradiation was defined as positive in patients who had undergone neck irradiation, including the area of the recipient vessel, prior to the actual surgery. The duration of surgery and the duration of flap ischemia were calculated as the length of time between the first incision and the last suture and that between the interruption of flap perfusion and the initiation of flap perfusion after anastomosis, respectively. Flap revision was defined as positive in patients who had undergone surgical revision of the anastomosis with return to the operating room, and flap success was defined as negative in patients in whom the flap had been removed because of flap necrosis. 

All surgical procedures were performed under general anesthesia with postoperative monitoring in the intensive care unit, with invasive mechanical ventilation and analgosedation until at least the next morning. Each patient’s blood pressure was monitored with an invasive arterial catheter and regulated by central venous vasopressor (norepinephrine) administration as needed, with a target systolic pressure above 125 mmHg.

### 2.2. Flap Perfusion Measurement Data

The measurement data for flap perfusion were obtained intraoperatively (immediately after anastomosis release) in the operating room and postoperatively (on the first postoperative morning) in the intensive care unit, using the O2C tissue oxygen analysis system (O2C Oxygen-to-see, LEA Medizintechnik, Giesen, Germany, software version 31.33), which has been described in previous studies [8,10,27]. Briefly, laser Doppler spectroscopy (830 nm; 30 mW) is used to determine the flap blood flow (arbitrary units [AU]) at 8 and 2 mm tissue depth, and white light spectroscopy (500–800 nm; 50 W) is used to determine the hemoglobin concentration (AU) and hemoglobin oxygen saturation (%) at 8 and 2 mm tissue depth [7,8]. The measurement probe was placed centrally on the dried skin portion of the flap in a sterile cover. The measurement time was 10 s (with a lead time of 4 s) under ambient light compensation control. 

### 2.3. Statistical Analysis

The patients were divided into groups according to the recipient vessels used for anastomosis. There were four groups for arterial anastomosis recipient vessels (ECA, FAA, LIA, and STA) and four groups for venous anastomosis recipient vessels (IJV, IJV + IJVB, IJVB, and EJV). In addition, the patients were also divided into two groups according to their American Society of Anesthesiologists score (ASA) (ASA > 2 and ASA ≤ 2). Differences in clinical parameters between the groups were analyzed using the chi-squared test or Freeman–Halton test for categorical data and the Kruskal–Wallis test for metric data. Between-group differences in perfusion measurement values (i.e., flap blood flow, hemoglobin concentration, and hemoglobin oxygen saturation) were analyzed with univariable testing using the Kruskal–Wallis test for all groups and, in the case of significant differences between all groups, the Mann–Whitney test for two groups. In addition, multivariable testing with multiple linear regression models was performed in the case of significant differences between groups. Multivariable testing for differences between arterial anastomosis recipient vessels was performed with adjustment for flap type (RFFF vs. ALTF vs. FFF), surgery duration, prior neck dissection status, prior neck irradiation status, mean arterial blood pressure (mmHg), and administered catecholamine dose (µg/min per kg). Values of *p* < 0.05 were considered statistically significant. The statistical analysis was conducted using SPSS version 28 (SPSS, IBM, New York, NY, USA). 

## 3. Results

### 3.1. Clinical Characteristics of the Study Population

The study population consisted of 338 patients (Table 2). Patients with different arterial anastomosis recipient vessels (i.e., ECA, FAA, LIA, or STA) differed in terms of flap type (*p* = 0.003), surgery duration (*p* = 0.004), prior neck dissection status (*p* < 0.001), and prior neck irradiation status (*p* < 0.001). Patients with different venous anastomosis recipient vessels (i.e., IJV, IJV + IJVB, IJVB, or EJV) differed in terms of flap type (*p* < 0.001) and location (*p* = 0.003). 

Patients with different arterial anastomosis recipient vessels (i.e., ECA, FAA, LIA, or STA) or different venous anastomosis recipient vessels (i.e., IJV, IJV + IJVB, IJVB, or EJV) did not differ in terms of flap success (ECA n = 24 (100.0%), FAA n = 132 (96.4%), LIA n = 20 (100.0%), STA n = 156 (99.4%), *p* = 0.288; and IJV n = 253 (98.4%), IJV + IJVB n = 41 (95.3%), IJVB n = 24 (100.0%), EJV n = 14 (100.0%), *p* = 0.415) or flap revision (ECA n = 1 (4.2%), FAA n = 10 (7.3%), LIA n = 0 (0.0%), STA n = 6 (3.8%), *p* = 0.472; and IJV n = 13 (5.1%), IJV + IJVB n = 3 (7.0%), IJVB n = 0 (0.0%), EJV n = 1 (7.1%), *p* = 0.529). 

### 3.2. Comparison of Flap Perfusion between Arterial Anastomosis Recipient Vessels

**Figure 1:**
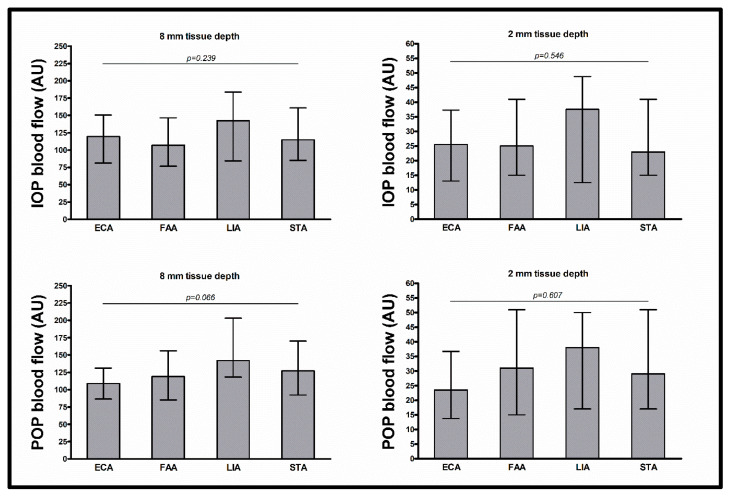
Intraoperative and postoperative blood flow. Data shown as median (with interquartile range) for blood flow (AU) (separately shown for intraoperative measurement at 8 mm tissue depth (upper left) and 2 mm tissue depth (upper right) and for postoperative measurement at 8 mm tissue depth (lower left) and 2 mm tissue depth (lower right)); *p*-values corresponding to testing for differences between arterial anastomosis recipient vessels with Kruskal–Wallis test (ECA vs. FAA vs. LIA vs. STA); abbreviations: ECA = external carotid artery, FAA = facial artery, LIA = lingual artery, STA = superior thyroid artery, IOP = intraoperative, POP = postoperative, AU = arbitrary units.

**Figure 2:**
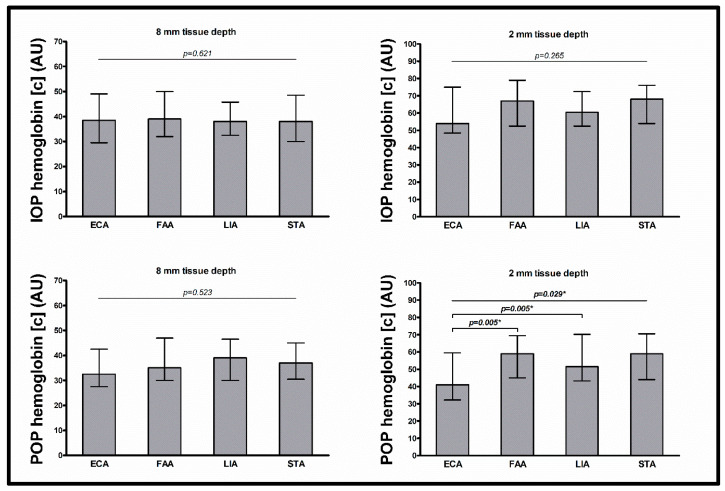
Intraoperative and postoperative hemoglobin concentration. Data shown as median (with interquartile range) for hemoglobin concentration (AU) (separately shown for intraoperative measurement at 8 mm tissue depth (upper left) and 2 mm tissue depth (upper right) and for postoperative measurement at 8 mm tissue depth (lower left) and 2 mm tissue depth (lower right)); *p*-values corresponding to testing for differences between arterial anastomosis recipient vessels with Kruskal–Wallis test (ECA vs. FAA vs. LIA vs. STA) and Mann–Whitney test (ECA vs. FAA; ECA vs. STA); significant *p*-values are bold (* *p* > 0.05 upon adjustment for flap type, surgery duration, prior neck dissection, prior neck irradiation, mean arterial blood pressure (mmHg), and administered catecholamine dose (µg/min per kg) in multiple linear regression analysis); abbreviations: ECA = external carotid artery, FAA = facial artery, LIA = lingual artery, STA = superior thyroid artery, IOP = intraoperative, POP = postoperative, AU = arbitrary units.

**Figure 3:**
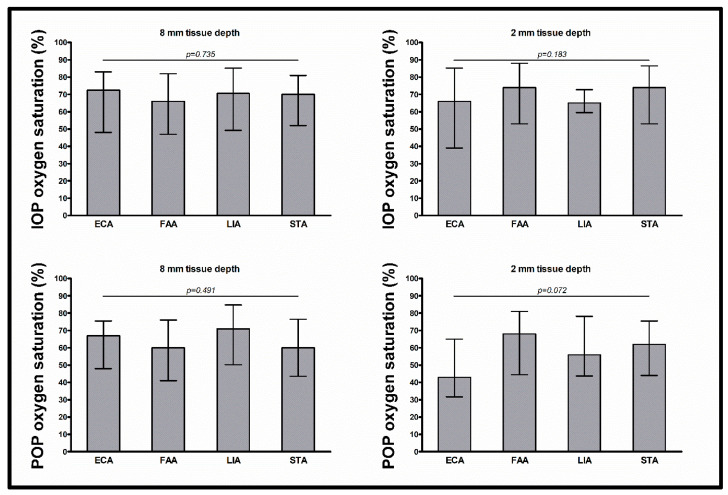
Intraoperative and postoperative hemoglobin oxygen saturation. Data shown as median (with interquartile range) for hemoglobin oxygen saturation (%) (separately shown for intraoperative measurement at 8 mm tissue depth (upper left) and 2 mm tissue depth (upper right) and for postoperative measurement at 8 mm tissue depth (lower left) and 2 mm tissue depth (lower right)); *p*-values corresponding to testing for differences between arterial anastomosis recipient vessels with Kruskal–Wallis test (ECA vs. FAA vs. LIA vs. STA); abbreviations: ECA = external carotid artery, FAA = facial artery, LIA = lingual artery, STA = superior thyroid artery, IOP = intraoperative, POP = postoperative.

The intraoperative flap blood flow, hemoglobin concentration, and hemoglobin oxygen saturation at tissue depths of 8 and 2 mm were similar between arterial anastomosis recipient vessels (all *p* > 0.05) (Table 3, Figure 1, Figure 2 and Figure 3). 

The postoperative flap blood flow and hemoglobin oxygen saturation at tissue depths of 8 and 2 mm and the postoperative hemoglobin concentration at a tissue depth of 8 mm were similar between arterial anastomosis recipient vessels (all *p* > 0.05). The postoperative hemoglobin concentration at a tissue depth of 2 mm differed between arterial anastomosis recipient vessels (*p* = 0.029), with lower values for the ECA than for the FAA and STA (41.0 AU vs. 59.0 AU, *p* = 0.005; and 41.0 AU vs. 59.0 AU; *p* = 0.005, respectively). In multivariable testing with adjustment for flap type (RFFF vs. ALTF vs. FFF), surgery duration, prior neck dissection status, prior neck irradiation status, mean arterial blood pressure, and administered catecholamine dose, the differences in postoperative hemoglobin concentration at a tissue depth of 2 mm between all arterial anastomosis recipient vessels, between the ECA and the FAA, and between the ECA and the STA, did not persist (*p* = 0.342, *p* = 0.245, and *p* = 0.083, respectively). In multivariable testing between all arterial recipient vessels, only flap type was a significant predictor (flap type (RFFF vs. ALTF vs. FFF) *p* < 0.001, surgery duration *p* = 0.286, prior neck dissection status *p* = 0.353, prior neck irradiation status *p* = 0.080, mean arterial blood pressure *p* = 0.389, and administered catecholamine dose *p* = 0.840). 

### 3.3. Comparison of Flap Perfusion between Venous Anastomosis Recipient Vessels

The intraoperative and postoperative flap blood flow, hemoglobin concentration, and hemoglobin oxygen saturation at tissue depths of 8 and 2 mm were similar between venous anastomosis recipient vessels (i.e., IJV, IJV + IJVB, IJVB, and EJV) (all *p* > 0.05) (Table 4). 

## 4. Discussion

This study investigated the influence of arterial and venous anastomosis recipient vessels on flap perfusion in microvascular head and neck reconstruction, as flap perfusion is essential for flap viability and thus flap survival and is therefore often used as a parameter for postoperative flap monitoring [7,8,9,10,11]. 

The knowledge of potential confounding variables such as the anastomosis recipient vessel is crucial for accurate postoperative flap monitoring based on flap perfusion [8,9,10]. In addition, the selection of arterial and venous anastomosis recipient vessels is one of the most important steps in microvascular free flap transfer since blood supply to the flap tissue is initially dependent on the anastomosis recipient vessels [3,4,6]. In general, the choice of the free flap type depends on the tissue requirements at the recipient site, which include skin, muscle or bone structures, while the choice of the anastomosis recipient vessels depends on several factors such as vessel location, vessel length, and vessel diameter [13]. In light of the fact that microvascular free flap failure is occasionally due to a gradual shutdown and not always due to an immediate complete interruption of microvascular free flap perfusion, the selection of anastomosis recipient vessels that ensure high flap blood inflow and outflow might be advantageous [3,22,23,24,28]. However, it remains unknown whether the anastomosis recipient vessels have an influence on microvascular free flap perfusion. 

Therefore, in this study, the influences of commonly used arterial (i.e., ECA, FAA, LIA, and STA) and venous anastomosis recipient vessels (i.e., IJV, IJV + IJVB, IJVB, and EJV) on microvascular flap perfusion were investigated using measurements with the Oxygen-to-see tissue oxygen analysis system [14,15]. Other methods for assessing flap perfusion used in other studies are based, for example, on tissue oxygen measurement, hydrogen clearance, microdialysis, or thermography [23]. This study neither investigated the influence of the choice of arterial or venous anastomosis recipient vessel on flap survival nor determined perfusion thresholds for flap failure, as these topics have been addressed in previous studies, and several potential confounding variables related to flap success were not covered by the data set of this study [8,10,18,25,29,30]. 

The results of this study demonstrated that intraoperative and early postoperative microvascular free flap perfusion—in terms of flap blood flow, hemoglobin concentration, and hemoglobin oxygen saturation at tissue depths of 8 and 2 mm—were similar between commonly used arterial anastomosis recipient vessels (i.e., ECA, FAA, LIA, and STA) and venous anastomosis recipient vessels or their combinations (i.e., IJV, IJV + IJVB, IJVB, and EJV). 

In univariable testing, the postoperative hemoglobin concentration at a tissue depth of 2 mm was lower for the ECA than it was for the FAA or the LIA. This could be due to group differences in flap type, as early postoperative hemoglobin concentration values differ between flap types [10]. In fact, the differences did not persist in multivariable testing with adjustment for between-group differences in flap type and duration of surgery, prior neck dissection status, and prior neck irradiation status as well as for presumed factors affecting flap perfusion, such as mean arterial blood pressure and catecholamine dose administered [31]. In general, the absence of differences in microvascular free flap perfusion between arterial anastomosis recipient vessels is remarkable in terms of the relationship between vessel diameter, resistance, and blood flow [20,21]. Studies have reported that the median vessel diameter of the ECA in the neck without prior surgery or irradiation is 70% larger than the vessel diameters of the FAA, LIA, and STA and, therefore, according to physiological principles, should promote blood flow because of lower vascular resistance [16,17]. Nevertheless, the postoperative flap blood flow at a tissue depth of 8 mm tended to be even lower for the ECA than for the FAA, LIA, and STA. A possible explanation for the lower flow values for the ECA could be a higher vascular scarring with vessel diameter reduction, since the ECA is predominantly used in our department in cases of unavailable FAA, LIA, or STA merely because of a prior neck dissection or microvascular reconstruction. With regard to the venous anastomosis recipient vessels and the respiratory pump effect, which is more pronounced in the IJV than in the EJV, the intraoperative flap blood values tended to be higher for the IJV, IJV + IJVB, and IJVB than for the EJV. This might be explained by a greater magnitude of the respiratory pump effect in the IJV, which supports venous flap drainage through a suction effect and consequently increases flap blood flow due to the venoarteriolar response [18,21,32]. 

With respect to comparable studies regarding the perfusion measurement method and flap types studied, flap blood flow, hemoglobin concentration, and hemoglobin oxygen saturation in the current study were comparable [33]. 

In general, the absence of differences in the perfusion of microvascular free flaps anastomosed to different recipient vessels with presumably distinct perfusion-determining conditions may indicate that under altered circulation conditions on the recipient side after anastomosis, free flap perfusion depends on the requirements of the free flap tissue rather than on the anastomosis recipient vessels [23,34,35]. 

This study has several limitations. Regarding the measurement method, it should be taken into account that a moist environment can complicate the measurement procedure; additionally, skin temperature affects skin perfusion [33,36]. However, all flaps were cleaned and dried before measurement. It should be noted that flap perfusion may have been influenced by factors not considered in this study (e.g., flap pedicle length), which consequently hampers the comparison of flap perfusion between patients and anastomosis recipient vessels, respectively. In addition, the measurement of flap perfusion and thus the inclusion of a patient in the study was only possible for patients with an at least initially successful transferred and perfused flap, which limits the generalizability of the study results. Furthermore, no preoperative reference values for flap perfusion before flap harvest were available because of the retrospective nature of the study. Most importantly, flap perfusion is critical to flap viability and success well beyond the short time frame examined in this study, and changes in flap perfusion in the longer term cannot be excluded [11,37]. 

Despite the fact that microvascular free flap survival is determined by the interplay of multiple factors in addition to adequate flap perfusion, the similar flap perfusion values observed between commonly used arterial and venous anastomosis recipient vessels might explain the indifferent success rates between these anastomosis recipient vessels reported in previous studies and also observed in this study population in the respect that comparable flap perfusion degrees may reflect comparable initial functionality of the technical procedure [7,8,9,11,12,29,30]. Interestingly, and supporting this assumption, in this study, blood flow and hemoglobin oxygen saturation were partially lower in failed flaps compared to successful flaps (intraoperative blood flow at 8 mm tissue depth 53.0 (67.8) vs. 116.0 (73.8), Mann–Whitney test *p* = 0.015; intraoperative hemoglobin oxygen saturation at 2 mm tissue depth 52.0 (36.5) vs. 73.0 (34.0), Mann–Whitney test *p* = 0.034; postoperative hemoglobin oxygen saturation at 2 mm tissue depth 16.0 (40.0) vs. 63.0 (35.8), Mann–Whitney test *p* = 0.002).

This study provides insight into microvascular free flap perfusion and demonstrates that flap perfusion initially appears to be independent of the arterial and venous anastomosis recipient vessels. It underlines the capability of the studied anastomosis recipient vessels to adequately perfuse free flaps, and this might explain the observed similar flap success rates between arterial and venous anastomosis recipient vessels. In terms of potential clinical implications, this study emphasizes the use of all studied recipient vessels for the free flap anastomosis in microvascular head and neck reconstruction. In addition, this study implies that the recipient vessel is not a confounding variable for flap monitoring with the Oxygen-to-see tissue analysis system based on flap perfusion with predefined threshold values indicating vascular flap compromise, i.e., absolute minimum values for flap blood flow and hemoglobin oxygen saturation [8,10]. However, with regard to the selection of the arterial and venous anastomosis recipient vessel, it will not change surgical practice, as the challenging step of selecting the most appropriate anastomosis recipient vessels to ensure optimal blood supply to the flap tissue with presumably beneficial high blood inflow and outflow cannot be guided by flap perfusion values in the absence of a difference between anastomosis recipient vessels [3,22,23,24,38]. Further studies are needed to confirm the results, in which a prospective study design with the possibility to control for confounding factors would be advantageous. 

## 5. Conclusions

This study demonstrates that the arterial and venous anastomosis recipient vessels have no influence on intraoperative and initial postoperative microvascular free flap perfusion, as flap blood flow, hemoglobin concentration, and hemoglobin oxygen saturation were similar between arterial and venous anastomosis recipient vessels. Indifferences in flap perfusion may explain the observed similar success rates observed for commonly used arterial and venous anastomosis recipient vessels in microvascular free flap reconstruction in the head and neck region and imply that the anastomosis recipient vessel is not a confounding variable in flap monitoring based on flap perfusion. 

## Figures and Tables

**Table 1:** Anastomosis parameters.

Recipient Vessel	Configuration	Number	Patient Number (%)
Arterial anastomosis *			
ECA	end-to-end	1	24 (7.1)
FAA	end-to-end	1	137 (40.5)
LIA	end-to-end	1	20 (5.9)
STA	end-to-end	1	157 (46.4)
Venous anastomosis **			
IJV	end-to-side	1 or 2	257 (76.0)
IJV + IJVB	end-to-side and end-to-end	2	43 (12.7)
IJVB	end-to-end	1	24 (7.1)
EJV	end-to-side or end-to-end	1 or 2	14 (4.1)

Description of anastomosis parameters in terms of anastomosis recipient vessel, anastomosis configuration, anastomosis number and patient number (with percentage) (separately described for arterial anastomosis and venous anastomosis); *p*-values corresponding to testing for differences between groups in terms of venous anastomosis recipient vessels (IJV vs. IJV + IJVB vs. IJVB vs. EJV) (* *p* = 0.446) and in terms of arterial anastomosis recipient vessels (ECA vs. FAA vs. LIA vs. STA) (** *p* = 0.441) with Freeman–Halton test; abbreviations: ECA = external carotid artery, FAA = facial artery, LIA = lingual artery, STA = superior thyroid artery, IJV = internal jugular vein, IJVB = internal jugular vein branch, EJV = external jugular vein.

**Table 2:** Study population.

Variable	All Patients (n = 338)	*p*-Value 1	*p*-Value 2
Sex (n)			
Male	173 (51.2%)	0.340	0.587
Female	165 (48.8%)		
Age (years)	64.0 (18.0)	0.563	0.777
BMI (kg/m^2^)	24.4 (6.1)	0.108	0.110
ASA (n)			
1 + 2	177 (52.4%)	0.077	0.986
3 + 4	161 (47.6%)		
Flap type (n)			
RFFF	160 (47.3%)	**0.003**	**0.001**
ALTF	133 (39.3%)		
FFF	45 (13.3%)		
Flap location (n)			
Tongue	46 (13.6%)	0.840	**0.003**
Floor of mouth	69 (20.4%)		
Mandible	93 (27.5%)		
Maxilla + hard palate	44 (13.0%)		
Cheek	26 (7.7%)		
Soft palate	15 (4.4%)		
Extraoral	45 (13.3%)		
Surgery duration (min)	547.5 (154.0)	**0.004**	0.191
Flap ischemia duration (min)	106.5 (35.0)	0.205	0.855
Diabetes (n)			
No	288 (85.2%)	0.906	0.069
Yes	50 (14.8%)		
Arterial hypertension (n)			
No	216 (63.9%)	0.805	0.735
Yes	122 (36.1%)		
Peripheral arterial disease (n)			
No	328 (97.0%)	0.885	0.801
Yes	10 (3.0%)		
Smoking status (n)			
No	206 (60.9%)	0.745	0.153
Yes	132 (39.1%)		
Prior neck dissection (n)			
No	265 (78.4%)	**<0.001**	0.895
Yes	73 (21.6%)		
Prior neck irradiation (n)			
No	294 (87.0%)	**<0.001**	0.630
Yes	44 (13.0%)		
Flap survival (n)			
No	6 (1.8%)	0.288	0.415
Yes	332 (98.2%)		
Flap revision (n)			
No	321 (95.0%)	0.472	0.529
Yes	17 (5.0%)		

Parameters are indicated as numbers (with percentage) for categorical data (sex, ASA, flap type, flap location, diabetes, arterial hypertension, peripheral arterial disease, smoking status, prior neck dissection, prior neck irradiation, flap survival, flap revision) or median (with interquartile range) for metric data (age, BMI, surgery duration, flap ischemia duration); *p*-values corresponding to testing for differences between groups in terms of arterial anastomosis recipient vessels (ECA vs. FAA vs. LIA vs. STA) (*p*-value 1) and venous anastomosis recipient vessels (IJV vs. IJV + IJVB vs. IJVB vs. EJV) (*p*-value 2) with chi-squared test (sex, ASA, arterial hypertension, smoking status, prior neck dissection (*p*-value 1), prior neck irradiation (*p*-value 1)), Freeman–Halton test (flap type, flap location, diabetes, peripheral arterial disease, prior neck dissection (*p*-value 2), prior neck irradiation (*p*-value 2), flap survival, flap revision), and Mann–Whitney test (age, BMI, surgery duration, flap ischemia duration); significant *p*-values are bold; abbreviations: BMI = body mass index, ASA = American Society of Anesthesiologists score, RFFF = radial free forearm flap, ALTF = anterolateral thigh flap, FFF = fibula free flap.

**Table 3:** Flap perfusion parameters for arterial anastomosis recipient vessels.

Variable	ECA	FAA	LIA	STA	*p*-Value
Intraoperative measurement					
8 mm tissue depth					
FLOW (AU)	119.5 (69.3)	107.0 (70.0)	142.5 (99.5)	115.0 (76.0)	0.239
HB (AU)	38.5 (19.5)	39.0 (18.0)	38.0 (13.3)	38.0 (18.5)	0.621
SO2 (%)	72.5 (35.0)	66.0 (35.0)	70.5 (36.0)	70.0 (29.0)	0.735
2 mm tissue depth					
FLOW (AU)	25.5 (24.3)	25.0 (26.0)	37.5 (36.3)	23.0 (26.0)	0.546
HB (AU)	54.0 (26.5)	67.0 (26.5)	60.5 (20.0)	68.0 (22.0)	0.265
SO2 (%)	66.0 (46.3)	74.0 (35.0)	65.0 (13.3)	74.0 (33.5)	0.183
Postoperative measurement					
8 mm tissue depth					
FLOW (AU)	109.0 (44.8)	119.0 (71.0)	142.0 (85.3)	127.0 (78.0)	0.066
HB (AU)	32.5 (15.0)	35.0 (17.0)	39.0 (16.5)	37.0 (14.5)	0.523
SO2 (%)	67.0 (27.5)	60.0 (35.0)	71.0 (34.5)	60.0 (33.0)	0.491
2 mm tissue depth					
FLOW (AU)	23.5 (23.0)	31.0 (36.0)	38.0 (33.0)	29.0 (34.0)	0.607
HB (AU)	41.0 (27.3)	59.0 (24.5)	51.5 (27.0)	59.0 (26.5)	**0.029** *
SO2 (%)	43.0 (33.3)	68.0 (36.5)	56.0 (34.5)	62.0 (31.5)	0.072

Parameters are indicated as median (with interquartile range) for intraoperative and postoperative measurement (separately described for arterial anastomosis recipient vessels); *p*-values corresponding to testing for differences between groups with Kruskal–Wallis test (ECA vs. FAA vs. LIA vs. STA); significant *p*-values are bold (* *p* = 0.342 upon adjustment for flap type, surgery duration, prior neck dissection, prior neck irradiation, mean arterial blood pressure (mmHg), and administered catecholamine dose (µg/min per kg) in multiple linear regression analysis); abbreviations: FLOW = blood flow, HB = hemoglobin concentration, SO2 = hemoglobin oxygen saturation, ECA = external carotid artery, FAA = facial artery, LIA = lingual artery, STA = superior thyroid artery, AU = arbitrary units.

**Table 4:** Flap perfusion parameters for venous anastomosis recipient vessels.

Variable	IJV	IJV + IJVB	IJVB	EJV	*p*-Value
Intraoperative measurement					
8 mm tissue depth					
FLOW (AU)	117.0 (82.5)	126.0 (51.0)	99.5 (58.5)	77.5 (51.5)	0.055
HB (AU)	39.0 (18.5)	36.0 (25.0)	35.0 (15.5)	37.0 (8.8)	0.411
SO2 (%)	69.0 (31.0)	70.0 (38.0)	71.0 (30.5)	57.5 (48.5)	0.388
2 mm tissue depth					
FLOW (AU)	24.0 (28.5)	25.0 (21.0)	23.5 (33.0)	15.5 (13.0)	0.251
HB (AU)	68.0 (24.0)	68.0 (18.0)	54.5 (30.5)	64.5 (39.5)	0.100
SO2 (%)	72.0 (33.0)	75.0 (41.0)	73.0 (36.8)	72.5 (39.5)	0.805
Postoperative measurement					
8 mm tissue depth					
FLOW (AU)	125.0 (75.5)	111.0 (75.0)	106.5 (66.5)	127.0 (82.8)	0.510
HB (AU)	37.0 (16.0)	35.0 (17.0)	31.0 (11.8)	37.0 (16.3)	0.200
SO2 (%)	62.0 (34.5)	61.0 (26.0)	67.5 (30.3)	48.0 (37.3)	0.436
2 mm tissue depth					
FLOW (AU)	31.0 (34.5)	29.0 (29.0)	21.0 (26.5)	32.5 (42.0)	0.767
HB (AU)	58.0 (25.5)	57.0 (25.0)	44.5 (25.3)	53.0 (40.3)	0.390
SO2 (%)	63.0 (35.5)	62.0 (38.0)	67.0 (38.8)	49.5 (39.5)	0.485

Parameters are indicated as median (with interquartile range) for intraoperative and postoperative measurement (separately described for venous anastomosis recipient vessels); *p*-values corresponding to testing for differences between groups with Kruskal–Wallis test (IJV vs. IJVC vs. IJVB vs. EJV); abbreviations: FLOW = blood flow, HB = hemoglobin concentration, SO2 = hemoglobin oxygen saturation, IJV = internal jugular vein direct, IJV + IJVB = combination of internal jugular vein and internal jugular vein branch, IJVB = internal jugular vein branch, EJV = external jugular vein, AU = arbitrary units.

## Data Availability

The raw data supporting the conclusions of this article will be made available by the authors on request.

## Abbreviations

ECA = external carotid artery; FAA = facial artery; LIA = lingual artery; STA = superior thyroid artery; IJV = internal jugular vein; IJVB = internal jugular vein branch; EJV = external jugular vein; AU = arbitrary units; RFFF = radial free forearm flap; ALTF = anterolateral thigh flap; FFF = fibula free flap; FLOW = blood flow; HB = hemoglobin concentration; SO2 = hemoglobin oxygen saturation.
